# Case of Tumor Originating in the Fauces

**Published:** 1871-04

**Authors:** Kelburne King

**Affiliations:** Surgeon of the Hull General Infirmary.


### ARTICLE VI.

### *Case of Tumour Originating in the Soft Palate, and Protruding into the Isthmus of the Fauces.*

REMOVED BY OPERATION.

By Kelburne King, M.D., F.R.C.S.,

SURGEON TO THE HULL GENERAL INFIRMARY.

On the 16th of September last, Charles E—— aged twenty-eight years, consulted me regarding a tumour of the throat, of which he gave me the following history. About three months before he first experienced a difficulty in swallowing, arising from a swelling in the roof of his mouth. This had not previously attracted his attention, but he admitted that it was of considerable size when he first observed it. From this time it increased continually, and he experienced great inconvenience in swallowing, and in respiration, especially during the night; his voice also was much affected, rendering it difficult to understand what he said.

On examination, a large tumour was found to occupy the whole left side of the soft palate, pushing the uvula over to the opposite side, and almost out of sight. It nearly filled up the whole isthmus of the fauces. It extended downwards along the left side, filling up the hollow between the pillars of the fauces as far as the eyes could follow it in the direction of the palato-pharyngeal fold. The tonsil was seen stretched over its lower portion. It was about thesize of an apple, and was of a globular form. On examination with the finger, its consistence was found to be hard, firm, and unyielding. It occupied the whole thickness of the left side of the soft palate, and could be felt terminating in a free, somewhat roughened margin behind the posterior nares; but it had no special attachment to the posterior edge of the hard palâte, and could be completely isolated from the nasal cavity. The finger could be readily passed round the projecting part of the tumour, over which the tonsil and the mucous membrane were tightly stretched; but the attachment of the broad base of the tumour to the outer wall of the pharynx, and parts beyond, could not be defined. The idea communicated to the finger was rather that of tight impaction than of absolute adhesion to the surrounding tissues. The tumour terminated below in the same way as above, in a roughened projection, round which the finger could be passed behind and below the anterior pillar of the fauces, on the outer side of the pharynx, external to the epiglottidean folds and the superior opening of the glottis. The boundaries of the tumour could thus be ascertained in all directions except one. Externally it was limited by the ascending ramus of the inferior maxilla. Internally it projected into the isthmus faucium, which it almost closed. Above it was incorporated with the soft palate, and projected upwards into the posterior nasal fossa; below, its free margin could be felt, distinctly projecting into the pharynx, and separated by a space from the opening of the glottis. Anteriorly it was limited by the palato-glossal fold and mucous membrane of the soft palate stretched over and pushed before it; posteriorly its internal limits were traceable by the forefinger along the outer surface of the pharynx; but no distinct answer could be given to the question as to how far its broad base extended in a backward and outward direction, where it must lie in close proximity to the parotid gland and to the carotid arteries. On making an external examination, there was nothing remarkable except a considerable projection in the hollow behind the jaw and below the ear, as if the parotid gland as pressed outwards.

The symptoms were felt to be urgent by the patient, and I therefore explained to him that unless some method could be found of removing the tumour, there was no other way of affording relief. He was quite convinced of the dangerous nature of the malady, and expressed himself as ready to submit to whatever operation might be proposed for his relief.

Eventually he was admitted into the infirmary, on September 27th. My first idea was to divide the inferior maxilla at the symphysis, and to remove the tumour by incisions made within the mouth, but the impossibility of defining its posterior connexions deterred me; ultimately I devised and performed the following operation:—I determined to get at the tumour by laying open the face from the angle of the mouth to a little above the angle of the jaw, then sawing through the ramus to ascertain whether sufficient space could be obtained to detach the tumour from its connexions; if not, to remove what portion of the body of the bone might be necessary, and, in case of the great vessels being implicated in the posterior connexions, to command the possible hæmorrage by first of all placing on the common carotid a ligature to be tightened at will. On October 15th the man was placed under the influence of chloroform, and the usual incision for ligature of the common carotid artery was made along the edge of the sterno-mastoid muscle. Here the first difficulty of the operation occurred. The patient could not be fully placed under the influence of chloroform, in consequence of the difficult respiration which it produced. The struggling which resulted, and the rapid respiration, made it very difficult to expose the artery. In opening the sheath a superficial vein was wounded; but, with a little patience, a double catgut ligature, preserved in carbolic oil, was passed round the vessel. This was not tightened, but left to the care of an assistant. An incision was then made from the angle of the mouth to the inferior maxilla, a little above the last molar tooth, and a ligature was placed on the dividend ends of the facial artery. The last two molars of the upper jaw were extracted, and the incision was carried across the masseter muscle as far as the posterior edge of the inferior maxilla. The masseter was then divided, and, a couple of arteries bleeding freely, I was about to place a ligature on them when I observed that, although the respiratory movements were regularly performed, no air entered the lungs. It was evident that the tumour had slipped down on the glottis, and prevented the admission of air. I pulled the tongue forward with artery forceps, but without benefit, and, after a moment's hesitation as to opening the larynx, I passed my finger through the opening made by extracting the two molars, and found that I could lift up the tumour and allow the admission of air. Suffocation was thus temporarily averted; but as soon as I removed my finger the tumor slipped down, and the entrance of air was prevented. Holding up the tumour with my left forefinger, I sawed through the inferior maxilla about an inch above the angle; and then forcibly drawing down the lower fragment, I found that I had plenty of room to introduce my fingers into the cavity of the mouth, and command the position of the tumour. The danger of immediate suffocation being thus averted, I divided the soft palate to the right of the uvula, detatched it from the hard palate, and thus obtained command of the upper portion of the tumour. I then made two incisions through the mucous membrane, one in front and one behind, enclosing the tonsil behind them; and, forcing my fingers behind the tumour, drew it out between the divided portions of the inferior maxilla. On my making a final incision to complete the removal, a terrific gush of blood took place. Holding a sponge firmly in the wound, I asked the gentlemen in charge of the ligature on the carotid to tighten it. Unfortunately the ligature gave way; and in attempting to pass another, the venous hæmorrhage, which had quite stopped, returned. This rendered the passing of the second ligature somewhat difficult, but it was finally accomplished, and the ligature drawn tight. Returning then to the wound in the face and throat, and removing the sponge, wihch had been firmly held in its place, I was gratified to find that the hæmorrhage was quite controlled. I then divided the lower portion of the mucous membrane, which was alone adherent, and completed the removal of the tumour and the superjacent mucous membrane, On examining the cut surface, no vessels of any size could be found divided, but a small artery on the right side of the soft palate, of course not commanded by the ligature on the left carotid in the neck, bled freely and required a ligature, which was easily applied. The wound was brought together by sutures, a compress was left to prevent venous hæmorrhage in the neck, and the operation completed.

On examining the tumour, it was found to be of a surely fibrous character, firm and homogeneous throughout. It was completely enucleated, having no firm adhesion to any of the surrounding tissues, not even to the mucous membrane which covered it and had seemed to be inseparable from it.

The following is the result of a microscopic examination, made by my friend Mr. Hendry, of this town:—"The tumour itself is decidedly *fibrous*, with abundant granules and nuclei. The mucous membrane of the velum and uvula is, on its anterior surface, healthy, with beautiful large pavement epithelia; on the posterior surface are innumerable cylindrical and cilated epithelia. Both surfaces are apparently healthy. The tonsil gland is filled with nuclei and lozenge-shaped epithelia, with fibre-cells. The small tumours attached to the tonsil were vesicles filled with a purulent-like matter, abounding with fibre-cells, inflammatory corpuscles, and a very large amount of cholestine, in crystalline plates."

On reviewing the operation, the first thing that struck me was, that while the fear of hæmorrhage was the danger most present to my mind in planning it, the real danger, that had nearly proved fatal in the performance, was suffocation. Though there was a most profuse gush of blood at the moment of the final incision of the tumour, I believe that it was occasioned by the vessels which no doubt freely supplied a structure of rapid growth, and that they probably would have been commanded without recourse to the ligature of the carotid. This, however, I could not know pre viously. I have been unable to find any record of a similar case; but if one in all respects similar should occur in my practice again, I would dispense with the preliminary ligature of the carotid.

The rest of this man's history is soon told. As almost all the soft palate was excised, and a large wound existed in the side of the throat, I gave directions that no food should be given by the mouth, and that he should have beef-tea freely administered by enemata. In the evening he looked wonderfully well; and the next morning (October 16th), having had a good night, he expressed himself as feeling comfortable. Ice was administered by the mouth, and he had another good night. On the 17th, he said that he could swallow the water from the melted ice, and was allowed milk and beef-tea, of which he partook freely. On the 18th, he had a restless night, and the face was somewhat swollen, but his general condition was satisfactory. On the 19th, the left side of the face was red and swollen, the left eyelids were puffy, and swallowing was difficult. The whole surface of the face was painted with styptic colloid; twenty minims of tincture of the muriate of iron were administered every three hours; the bowels, which had not acted since the morning of the operation, were relieved by an enema; and afterwards the administration of beef-tea in this way was recommenced. On the 20th, the parts of the face which had been covered by the styptic colloid were considerably relieved, but the erysipelas had extended to the neck, where large bullæ were formed; his general condition was much as on the day before. The remedies were persevered with, and champagne taken freely by the mouth. On the morning of the 21st, while there was but little if any extension of the erysipelas, his general condition was more depressed, his pulse was weaker, and he seemed to take less notice of what was going on about him. In the afternoon and evening the symptoms of sinking became more marked, his extremities getting cold and the tendency to restlessness increasing and he died at 9.20 P. M.

In considering this result, it must, I think, be admitted that death arose, not from any necessary consequence of the operation, but from an accident. There were one or two cases of erysipelas in the house at the time. I was myself unfortunately in attendance on a very disastrous case, which, commencing on October 13th, terminated fatally on the 17th. This man had survived the immediate results of the operation; the power of swallowing was restored; the wound in the face had all but healed; the division of the inferior maxilla could not be looked upon as hazardous; no secondary hæmorrhage had taken place; from leaving the operating table till his death he did not lose one drop of blood. All the legitimate dangers, except those in connexion with the separation of the ligature on the common carotid, had been surmounted; and where so many and so great difficulties had been overcome, it seems all the more to be regretted that death should have arisen from a cause which a better acquaintance with physical laws will some day enable us to avoid, and which was foreign to the peculiar risks entailed by this very formidable operation.—*London Lancet.*

